# A 10-Year Review of Methotrexate Treatment for Ectopic Pregnancy in a Malaysian Tertiary Referral Hospital

**DOI:** 10.7759/cureus.30395

**Published:** 2022-10-17

**Authors:** Ahmad Akram Omar, Lua Khai Leng, Aruku Naidu Apana, Adibah Ibrahim, Rahimah Abdul Rahim, Najib Majdi Yaacob, Engku Ismail Engku-Husna

**Affiliations:** 1 Department of Obstetrics and Gynaecology, School of Medical Sciences, Universiti Sains Malaysia, Kubang Kerian, MYS; 2 Department of Obstetrics and Gynaecology, Hospital Raja Permaisuri Bainun, Ipoh, MYS; 3 Biostatistics and Research Methodology Unit, School of Medical Sciences, Universiti Sains Malaysia, Kubang Kerian, MYS

**Keywords:** transvaginal sonography, serum β-hcg, fetal cardiac activity, single-dose methotrexate protocol, ectopic pregnancy

## Abstract

Background

Ectopic pregnancy was recorded as the fourth principal cause of maternal death in Malaysia in 2019. Early diagnosis and use of methotrexate treatment proved to be safe and effective alternatives to surgical treatment. This study investigates the success rate of methotrexate treatment for ectopic pregnancy in a tertiary hospital in Malaysia.

Methods

This was a retrospective review of 73 patients with ectopic pregnancies treated with methotrexate according to a single-dose protocol from January 2009 until November 2019. The diagnosis of ectopic pregnancy was made using a combination of transvaginal scan and serial serum β-hCG levels. Their clinical and demographic data were reviewed. Serum β-hCG levels were measured at pre- and post-treatment to determine the rate of successful resolution.

Results

The overall success rate was 87.7% (64/73 patients) with methotrexate treatment. Fifty-six patients (76.7%) were successfully treated with a single dose of methotrexate, and eight patients (11.0%) required a second dose of methotrexate. There was no relation between socio-demographic, pre-treatment β-hCG levels, ectopic mass size, and treatment efficacy. Smaller size of ectopic pregnancy (adjusted OR=29.23; 95% CI: 2.69, 317.90; P=0.006) and absence of free fluid at the pouch of Douglas (POD) (adjusted OR=27.31; 95% CI: 2.84, 262.32; P=0.004) was found to increase the likelihood of overall treatment success. Absence of fetal cardiac activities was found to increase the likelihood of first-dose methotrexate treatment success (OR=10.20; 95% CI: 1.93, 53.79; P=0.006).

Conclusions

Early diagnosis of ectopic pregnancy may reduce morbidity and mortality. In carefully selected cases, methotrexate treatment has been proven to be cost-effective and avoided risks associated with surgery and anaesthesia.

## Introduction

Ectopic pregnancy (EP) is the leading cause of maternal mortality in the first trimester and accounts for 10%-15% of all maternal deaths [1]. With advances in scientific, laboratory, and imaging technologies, EP can be diagnosed at an early stage using transvaginal ultrasonography (TVS) and serum beta-human chorionic gonadotropin (β-hCG) assays [2,3].

The growth rate of the gestational sac is approximately 1.1 mm/day and the gestational sac first becomes apparent on TVS at approximately 4.5-5 weeks of gestational age, appearing as a round anechoic structure located eccentrically within the echogenic decidua [4]. The positive identification of a non-cystic adnexal mass with an empty uterus has a sensitivity of 84-90% and a specificity of 94-99% for the diagnosis of an ectopic gestation [5]. Suspicion of an ectopic pregnancy increases if free fluid (representing blood) is visualized, either surrounding the uterus or in the pouch of Douglas (POD), although a small amount of free fluid in the POD, a transudate due to increased vascular permeability, is common in early pregnancy [6].

The treatment for ectopic pregnancy can be expectant, medical, or surgical. The overall success rate of medical treatment in properly selected women is nearly 90% [7]. Previously, the standard treatment for managing ectopic pregnancy was surgery with a laparotomy approach. In current practice, laparoscopy has been used in the diagnosis of ectopic pregnancy for many years, and is being used with increasing frequency in the surgical treatment of ectopic pregnancy [8].

Nowadays, medical treatment of ectopic pregnancy with methotrexate (MTX) is preferred, as the need for surgery along with its associated complications is avoided. MTX treatment is also easier to manage and is more cost-effective than surgery. Moreover, MTX treatment has shown comparable success rates, safety, and fertility preservations with surgery, since single-dose and multi-dose MTX therapy protocols have been developed. The optimal treatment protocol has been discussed extensively in the literature [9-11], and success rates of single-dose and multiple-dose MTX regimens are similar [12]. Treatment with MTX, a folic acid antagonist highly toxic to rapidly replicating tissues, achieves results comparable to surgery for the treatment of appropriately selected ectopic pregnancies and is used commonly [13].

The success rate of medical treatment with MTX has been reported as between 75% and 96% in properly selected patients. Although both regimens have been studied extensively, there is no consensus on the optimum protocol. In a large meta-analysis of 1327 women, the success rate of single-dose was less than multi-dose regimen, without any significant difference (88% vs. 93%) [12]. National Institute for Health and Care Excellence (NICE) recommends that MTX should be the first-line management for women who are able to return for follow-up and who have no significant pain, an unruptured ectopic pregnancy with a mass smaller than 35 mm with no visible heartbeat, a serum β-hCG less than 1500 IU/L, and no intrauterine pregnancy (as confirmed on ultrasound scan) [14]. MTX also does not adversely impact ovarian reserve or subsequent fertility [15].

MTX is most often administered as a single intramuscular dose of 50 mg/m^2^, followed by measurement of serum β-hCG levels on days four and seven after MTX. If the β-hCG level decreases by more than 15%, then β-hCG levels are measured weekly until they are less than 15 IU/L. If the β-hCG level does not reduce by 15%, then administration of a second dose of MTX may be considered after a repeated ultrasound to exclude the presence of ectopic fetal cardiac activity and significant hemoperitoneum [16]. When β-hCG levels increase despite multiple doses of MTX or symptomatic or hemodynamic instability, surgical intervention is necessary. This study investigates the predictive factors of successful MTX treatment for ectopic pregnancy in a tertiary hospital in Malaysia.

## Materials and methods

Study design and setting

This retrospective study was carried out at the Hospital Raja Permaisuri Bainun (HRPB), Ipoh, from 1st January 2009 until 30th November 2019. This study was approved by the Medical Research & Ethics Committee (MREC) NMRR-19-1783-48974, and Human Research Ethics Committee University Sains Malaysia (HREC) USM/JEPeM/19070430.

Participants

Cases were diagnosed with an ectopic pregnancy at any stage of gestation, or ectopic pregnancy treated with MTX treatment in the Obstetrics & Gynecology (O&G) Department of HRPB, Ipoh, Malaysia. Cases of non-tubal ectopic pregnancy, such as cesarean scar pregnancy, and cornual and cervical ectopic pregnancies, were recruited into the study. These unusual sites of implantation were often diagnosed late (around 8-12 weeks gestation) compared with tubal pregnancy due to the distensibility of surrounding myometrial tissue, thus accommodating a larger gestational sac. An ultrasound scan mostly revealed a gestational sac with a fetal echo or measurable crown-rump length and positive fetal cardiac activity. Selection criteria for MTX included hemodynamically stable women, asymptomatic or with no significant pain, who were willing to attend follow-up, and no known hypersensitivity to MTX.

Data sources/measurement

The diagnostic workup for tubal and non-tubal ectopic pregnancy in the HPRB, Ipoh, is consistent with the Royal College of Obstetricians & Gynecologists, Green-top Guidelines No. 21 [14] and includes serial serum β-hCG measurements and transvaginal ultrasound scanning. There were two different brands of ultrasound machines used in the O&G department at HRPB, Ipoh: the Mindray ultrasound machine (Mahwah, NJ) and the GE Healthcare ultrasound machine (Chicago, IL). Both were attached together with an abdominal probe and an endovaginal probe. The endovaginal probe has frequencies that vary in the range of 5 MHz-9 MHz.

Serum β-hCG levels were then measured on day one (pre-treatment) and days four and seven of treatment. If the serum β-hCG levels declined by more than 15% between days four and seven, serum β-hCG levels were measured weekly until they were less than 15 IU/L. If the serum β-hCG level failed to decline by at least 15% between days four and seven, then a repeat dose of MTX was given. Repeat or second-dose MTX was administered intramuscularly. A successful response to MTX treatment was defined as the resolution of the β-hCG level to less than 15 IU/L without surgical intervention. Patients who underwent surgical treatment as the first option of management for ectopic pregnancy, or case notes with incomplete data were excluded.

Statistical analysis

Statistical analysis was performed with the Statistical Package for Social Sciences (SPSS) version 26.0 software (IBM Corp., Armonk, NY). Descriptive statistics were used to summarize the characteristics of the study participants. All numerical variables with Gaussian distribution were characterized by mean and standard deviation (SD), and those with non-Gaussian distribution were characterized by median and interquartile range (IQR). All categorical variables were characterized by frequency (n) and percentage (%).

Quantitative Variables

To determine factors associated with MTX treatment success, univariable (simple) and multivariable (multiple) binary logistic regression analyses were conducted. In the univariable analysis, variables with P<0.25 were included for variable selection of multivariable analysis. The forward and backward likelihood ratio (LR) method of variable selection was conducted for variable selection to obtain the preliminary main effect model. All retained variables were checked for interaction and multicollinearity to obtain the preliminary final model. The fitness of the obtained model was evaluated using the Hosmer-Lemeshow test, classification table, and area under the receiver operating characteristics (ROC) curve. The adjusted odds ratio (adjusted OR), the 95% confidence interval for the OR, and the P-value for the final model were presented. A P-value of <0.05 is considered statically significant in the final model. Treatment failures were defined as the need to undergo surgical intervention for any reason after MTX administration.

Bias

Three forms of potential bias in cohort studies were identified and handled in this study: 1) selection bias, 2) information bias, and 3) confounders and interaction. Selection bias is minimized by recruiting all ectopic pregnancy patients from only one center (HRPB Ipoh, Malaysia), as different treatment centers may have a different treatment protocol for the management of ectopic pregnancies. Information bias is minimized by evaluating objective measures of the treatment success (resolution of the β-hCG level to less than 15 IU/L without surgical intervention). Confounding and interaction effects of factors potentially associated with the treatment success were evaluated with multivariable statistical analysis.

Study Size

The sample size for this study was determined based on calculation for logistic regression analysis using G*Power Software version 3.1.9.7 [17]. A previous study on the predictive factors of MTX treatment success in ectopic pregnancy reported that the absence of fluid in the abdomen was an important predictor of treatment success. The proportion of patients with abdominal fluid was 37%, and the rate of success was 87.3% in the non-fluid and 62.2% in the fluid group (Odds Ratio = 4.18) [18]. For an Odds Ratio of 4.3 (large effect size) to be significant with 5% Type I error and 80% power of the study, the required sample size was 107 patients. Given the limited number of ectopic pregnancy patients in the study center throughout the entire 10 years period (from 2009 to 2019), all eligible patients were included in the study.

## Results

**Figure 1:**
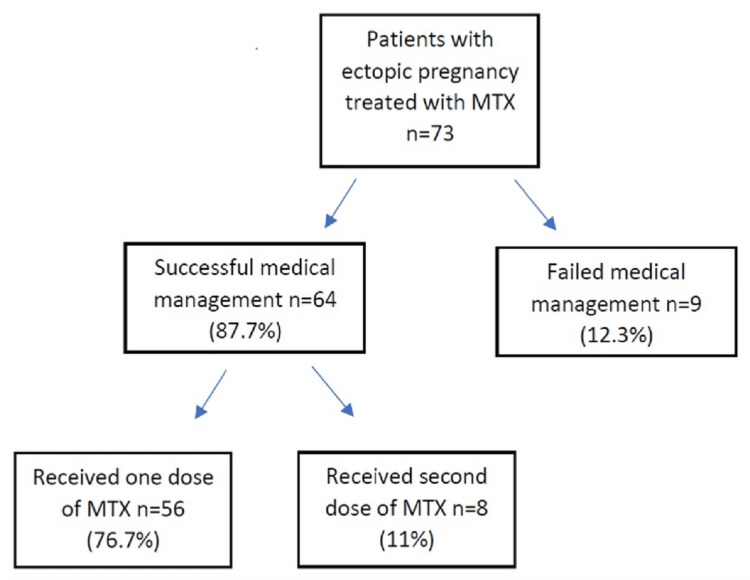
Flow diagram of patients with ectopic pregnancy treated with MTX MTX: Methotrexate

The overall success rate of MTX treatment was 87.7% (64/73), and eight patients (11%) required a second dose of MTX to achieve complete resolution. There were nine patients in whom treatment failed (12.3%, 9/73) (Figure 1).

The baseline data for the successful and failed treatment groups are shown in Table 1. There was no statistically significant difference in the sociodemographic characteristics of the groups.

**Table 1:** Characteristics of patients with ectopic pregnancies treated with methotrexate (N=73) * Median (IQR). For gestational age, size of ectopic, baseline β-hCG level skewed to the right. POD: Pouch of Douglas; MTX: Methotrexate; β-hCG: beta-human Chorionic Gonadotropin; BTL: Bilateral Tubal Ligation; IUCD: Intrauterine Contraceptive Device; C-Section: Cesarean section delivery.

Characteristic	Mean (SD)	n (%)
Sociodemographic		
Age (years)	30.67 (5.15)	
Age group (years)		
< 25		7 (9.6)
25 – 29		26 (35.6)
30 – 35		25 (34.2)
> 35		15 (20.5)
Ethnicity		
Malay		47 (64.4)
Chinese		11 (15.1)
Indian		9 (12.3)
Others		6 (8.2)
Marital status		
Married		71 (97.3)
Not married		2 (2.7)
Occupation		
Employed		50 (68.5)
Self-employed		6 (8.2)
Unemployed		17 (23.3)
Current obstetric history		
Gravida		
1		16 (21.9)
2		25 (34.2)
3		8 (11.0)
4		10 (13.7)
> 5		14 (19.2)
Parity		
0		28 (38.4)
1		22 (30.1)
2		11 (15.1)
3		11 (15.1)
4		1 (1.4)
Gestational age (weeks)	7.00 (2.00) *	
Location of ectopic pregnancy		
Tubal		48 (65.8)
Cornual		5 (6.8)
Cervical		3 (4.1)
Cesarean scar		17 (23.3)
Size of ectopic pregnancy (cm)	2.11 (0.89) *	
Size of ectopic pregnancy		
< 3.5 cm		64 (87.7)
> 3.5 cm		9 (12.3)
Yolk sac		
Absent		40 (54.8)
Present		33 (45.2)
Fetal cardiac activities		
Absent		61 (83.6)
Present		12 (16.4)
Free fluid at POD		
Absent		56 (76.7)
Present		17 (23.3)
Route of MTX		
Intramuscular		52 (71.2)
Transvaginal / Transcervical		20 (27.4)
Laparoscopic		1 (1.4)
Baseline b-hCG	2449.00 (6056.00) *	
Baseline b-hCG level		
< 1500		10 (13.7)
1500 – 4999		42 (57.5)
5000 – 9999		5 (6.8)
10,000 – 100,000		13 (17.8)
> 100,000		3 (4.1)
Past obstetric history		
Previous abortion		
No		45 (61.6)
Yes		28 (38.4)
Previous ectopic pregnancy		
No		63 (86.3)
Yes		10 (13.7)
Post BTL		
No		73 (100.0)
Yes		0 (0.0)
IUCD usage		
No		72 (88.6)
Yes		1 (1.4)
History of infertility		
No		67 (91.8)
Yes		6 (8.2)
Past gynecology history		
Previous non-tubal abdominal surgery		
No		71 (97.3)
Yes		2 (2.7)
Previous pelvic surgery		
No		71 (97.3)
Yes		2 (2.7)
Previous tubal surgery		
No		64 (87.7)
Yes		9 (12.3)
History of C-Section		
No		48 (65.8)
Yes		25 (34.2)
Number of C-section		
0		48 (65.8)
1		15 (20.5)
2		9 (12.3)
3		1 (1.4)

The most common age group of ectopic pregnancy cases who received MTX treatment was 25-35 years. In this study, the majority of patients reviewed were of Malay ethnicity (64.4%), followed by Chinese (15.1%) and Indian (12.3%); 28 (38.4%) of the patients were primigravida, and the remaining 45 (61.6%) were multigravida.

In the study, 28 (38.4%) of patients with ectopic pregnancy had suffered a previous miscarriage, and 25 (34.2%) had a history of cesarean section, which contributed to the increased incidence of cesarean scar pregnancy. Recurrent ectopic pregnancies were observed in 10 patients (13.7%). Nine (12.3%) patients had a history of tubal surgeries, while a history of subfertility with a duration of subfertility of more than five years was observed in six patients (8.2%). However, 27 patients (37%) had no identifiable risk factors for ectopic pregnancy.

The fallopian tube was the most common site for ectopic pregnancies (48 [65.8%] cases), followed by the cesarean scar (17 [23.3%] cases), which is associated with the increasing rate of cesarean sections performed in Malaysia and worldwide. There were five (6.8%) and three cases of ectopic pregnancy located in the cornual and cervical sites, respectively.

Patients with a diagnosis of ectopic pregnancy and planned medical treatment (n=73) were treated with a single dose of 50 mg/m^2^ MTX, which was administered either systemically or locally. In this study, systemic MTX was administrated intramuscularly, while local MTX was administrated directly into the ectopic gestational sac with transvaginal ultrasound guidance.

A majority of the patients (56 out of 73; 76.7%) successfully responded to the first dose of MTX treatment. Eight patients (11.0%) required a second dose of MTX, and all these patients responded well to the treatments given. Only nine patients (12.3%) failed MTX treatment and required surgical intervention, either laparoscopically or a laparotomy.

A univariable (simple) binary logistic regression analysis to determine factors associated with MTX treatment success is shown in Table 2.

**Table 2:** Simple logistic regression analysis to determine factors associated with methotrexate treatment success (N=73) POD: Pouch of Douglas; MTX: Methotrexate; β-hCG: beta-human Chorionic Gonadotropin; C-Section: Cesarean section delivery.

Characteristic	Fail, n=9 n (%)	Successful, n=64 n (%)	Crude OR (95% CI)	P-value
Sociodemographic				
Age (years)	29.00 (5.17)	30.91 (5.14)	1.08 (09.4, 1.24)	0.300
Ethnicity				
Malay	5 (10.6)	42 (89.4)	Ref	
Non-Malay	4 (15.4)	22 (84.6)	0.66 (0.16, 2.69)	0.557
Current obstetric history				
Gravida				
1	1 (6.3)	15 (93.8)	Ref	
2	4 (16.0)	21 (84.0)	0.35 (0.04, 3.45)	0.369
3	1 (12.5)	7 (87.5)	0.47 (0.03, 8.60)	0.608
4	1 (10.0)	9 (90.0)	0.60 (0.03, 10.82)	0.729
> 5	2 (14.3)	12 (95.7)	0.40 (0.03, 4.96)	0.476
Parity				
0	4 (14.3)	24 (85.7)	Ref	
1	2 (9.1)	20 (90.9)	1.67 (0.28, 10.06)	0.578
2	1 (9.1)	10 (90.2)	1.67 (0.17, 16.83)	0.665
3 or more	2 (16.7)	10 (83.3)	0.83 (0.13, 5.30)	0.847
Gestational age (weeks)	7.33 (1.66)	7.26 (2.13)	0.98 (0.71, 1.37)	0.926
Location of ectopic pregnancy				
Tubal	7 (14.6)	41 (85.4)	Ref	
Cesarean scar	2 (11.8)	15 (88.2)	1.28 (0.24, 6.86)	0.773
Cornual or Cervical	0 (0.0)	8 (100.0)	Unable to calculate	
Size of ectopic pregnancy				
< 3.5 cm	4 (6.3)	60 (93.8)	18.75 (3.57, 98.54)	0.001
> 3.5 cm	5 (55.6)	4 (44.4)	Ref	
Yolk sac				
Absent	0 (0.0)	40 (100.0)	Ref	
Present	9 (27.3)	24 (72.7)	Unable to calculate	
Fetal cardiac activities				
Absent	6 (9.8)	55 (90.2)	3.06 (0.65, 14.47)	0.159
Present	3 (25.0)	9 (75.0)	Ref	
Free fluid at POD				
Absent	2 (3.6)	54 (96.4)	18.90 (3.42, 104.52)	0.001
Present	7 (41.2)	10 (58.8)	Ref	
Route of MTX				
Intramuscular	7 (13.5)	45 (86.5)	Ref	
Transvaginal/ Transcervical	2 (10.0)	18 (90.0)	1.40 (0.27, 7.39)	0.692
Laparoscopic	0 (0.0)	1 (100.0)	Unable to calculate	
Baseline b-hCG level				
< 1500	1 (10.0)	9 (90.0)	Ref	
1500 – 4999	5 (11.9)	37 (88.1)	0.82 (0.09, 7.94)	0.866
5000 – 9999	1 (20.0)	4 (80.0)	0.44 (0.02, 9.03)	0.598
> 10,000	2 (12.5)	14 (87.5)	0.78 (0.06, 9.89)	0.846
Past obstetric history				
Previous abortion				
No	6 (13.3)	39 (86.7)	0.78 (0.18, 3.41)	0.741
Yes	3 (10.7)	25 (89.3)	Ref	
Previous ectopic pregnancy				
No	7 (11.1)	56 (88.9)	2.00 (0.35, 11.36)	0.434
Yes	2 (20.0)	8 (80.0)	Ref	
History of infertility				
No	7 (10.4)	60 (89.6)	4.29 (0.66, 27.79)	0.127
Yes	2 (33.3)	4 (66.7)	Ref	
Past gynecology history				
Previous tubal surgery				
No	7 (10.9)	57 (89.1)	2.33 (0.40, 13.48)	0.346
Yes	2 (22.2)	7 (77.8)	Ref	
History of C-Section				
No	7 (14.6)	41 (85.4)	0.51 (0.10, 2.66)	0.424
Yes	2 (8.0)	23 (92.0)	Ref	

Among 10 patients with pre-treatment β-hCG levels below 1500 IU/L, nine (90%) were successfully treated, and among 47 patients with pre-treatment β-hCG levels between 1500 IU/L to 100,000 IU/L, 41 (87.2%) were successfully treated. Among patients with pre-treatment β-hCG levels of >10,000, 14 (87.5%) were successfully treated. At univariable analysis, the size of ectopic pregnancy and the presence of free fluid at the POD were found to be significant factors associated with treatment success. Two other variables (fetal cardiac activities and history of infertility) were included in the variable selection of multivariable (multiple) binary logistic regression analysis. The final model of multiple logistic regression analysis indicated that only two variables were significantly associated with treatment success (Table 3).

**Table 3:** Multiple logistic regression analysis to determine factors associated with methotrexate treatment success (N=73) POD: Pouch of Douglas Size of ectopic, cardiac activities, free fluid, and history of infertility were included for variable selection using the backward likelihood ratio (LR) variable selection method. Only the size of ectopic and free fluid is retained in the preliminary main effect model. No interaction and no multicollinearity between the two variables. Assessment of model fitness: Hosmer Lemeshow test Chi2 (1) = 0.25, p = 0.615; Classification table overall percentage of correct prediction = 93.2%; Area under ROC curve = 88.2% (95% CI: 74.5, 100.0).

Characteristic	Crude OR (95% CI)	P-value	Adjusted OR (95% CI)	P-value
Size of ectopic pregnancy				
< 3.5 cm	18.75 (3.57, 98.54)	0.001	29.23 (2.69, 317.90)	0.006
> 3.5 cm	Ref		Ref	
Free fluid at POD				
Absent	18.90 (3.42, 104.52)	0.001	27.31 (2.84, 262.32)	0.004
Present	Ref		Ref	

Smaller size of ectopic pregnancy (adjusted OR=29.23; 95% CI: 2.69, 317.90; P=0.006) and absence of free fluid at the POD (adjusted OR=27.31; 95% CI: 2.84, 262.32; P=0.004) were found to increase the likelihood of treatment success.

Univariable (simple) and multivariable (multiple) binary logistic regression analyses to determine factors associated with first-dose MTX treatment success are presented in Tables 4, 5.

**Table 4:** Simple logistic regression analysis to determine factors associated with first-dose methotrexate treatment success (N=64) POD: Pouch of Douglas; MTX: Methotrexate; β-hCG: beta-human Chorionic Gonadotropin; C-Section: Cesarean section delivery.

Characteristic	Success second dose (n=8) n (%)	Success first dose MTX (n=56) n (%)	Crude OR (95% CI)	P-value
Sociodemographic				
Age (years)	31.88 (6.31)	30.77 (5.01)	0.96 (0.83, 1.11)	0.567
Ethnicity				
Malay	5 (11.9)	37 (88.1)	Ref	
Non-Malay	3 (13.6)	19 (86.4)	0.66 (0.16, 2.69)	0.557
Current obstetric history				
Gravida				
1	2 (13.3)	13 (86.7)	Ref	
2	1 (4.8)	20 (95.2)	3.08 (0.25, 37.48)	0.378
3	2 (28.6)	5 (71.4)	0.39 (0.04, 3.52)	0.385
4	0 (0.0)	9 (100.0)	Unable to calculate	-
> 5	3 (25.0)	9 (75.0)	0.46 (0.06, 3.35)	0.444
Parity				
0	3 (12.5)	21 (97.5)	Ref	
1	2 (10.0)	18 (90.0)	1.29 (0.19, 8.57)	0.795
2	2 (20.0)	8 (80.0)	0.57 (0.08, 4.08)	0.577
3 or more	1 (10.0)	9 (90.0)	1.29 (1.12, 14.09)	0.837
Gestational age (weeks)	7.75 (3.20)	7.20 (1.97)	0.90 (0.66, 1.27)	0.493
Location of ectopic pregnancy				
Tubal	4 (9.8)	37 (90.2)	Ref	
Cesarean scar	2 (13.3)	13 (86.7)	0.70 (1.12, 4.30)	0.703
Cornual or Cervical	2 (25.0)	6 (75.0)	0.32 (0.05, 2.18)	0.246
Size of ectopic pregnancy				
< 3.5 cm	7 (11.7)	53 (88.3)	2.52 (0.23, 27.72)	0.449
> 3.5 cm	1 (25.0)	3 (75.0)	Ref	
Yolk sac				
Absent	2 (5.0)	38 (95.0)	6.33 (1.16, 34.52)	0.033
Present	6 (25.0)	18 (75.0)	Ref	
Fetal cardiac activities				
Absent	4 (7.3)	51 (92.7)	10.20 (1.93, 53.79)	0.006
Present	4 (44.4)	5 (55.6)	Ref	
Free fluid at POD				
Absent	6 (11.1)	48 (88.9)	2.00 (0.34, 11.70)	0.442
Present	2 (20.0)	8 (80.0)	Ref	
Route of MTX				
Intramuscular	4 (8.9)	41 (91.1)	ref	
Transvaginal/ Transcervical	3 (16.7)	15 (83.3)	0.48 (0.10, 2.44)	0.382
Laparoscopic	1 (100.0)	0 (0.0)	Unable to calculate	
Baseline b-hCG level				
< 1500	1 (11.1)	8 (88.9)	Ref	
1500 – 4999	3 (8.1)	34 (91.9)	1.14 (0.13, 15.47)	0.775
5000 – 9999	1 (25.0)	3 (75.0)	0.38 (0.02, 8.10)	0.532
> 10,000	3 (21.4)	11 (78.6)	0.46 (0.04, 5.27)	0.531
Past obstetric history				
Previous abortion				
No	3 (7.7)	36 (92.3)	3.00 (0.65, 13.89)	0.160
Yes	5 (20.0)	20 (80.0)	Ref	
Previous ectopic pregnancy				
No	8 (14.3)	48 (85.7)	Unable to calculate	-
Yes	0 (0.0)	8 (100.0)	Ref	
History of infertility				
No	7 (11.7)	53 (88.3)	2.52 (0.23, 27.72)	0.449
Yes	1 (25.0)	3 (75.0)	Ref	
Past gynecology history				
Previous tubal surgery				
No	8 (14.0)	49 (86.0)	Unable to calculate	-
Yes	0 (0.0)	7 (100.0)	Ref	
History of C-Section				
No	6 (14.6)	35 (85.4)	0.56 (0.10, 3.01)	0.495
Yes	2 (8.7)	21 (91.3)	Ref	

**Table 5:** Multiple logistic regression analysis to determine factors associated with methotrexate treatment success (N=73) Yolk sac, cardiac activities, and history of previous abortion were included for variable selection using the backward likelihood ratio (LR) variable selection method. Only cardiac activities are retained in the preliminary main effect model. Assessment of model fitness: Classification table overall percentage of correct prediction = 87.5%; Area under ROC curve = 76.1% (95% CI: 56.3, 96.0).

Characteristic	Crude OR (95% CI)	P-value	Adjusted OR (95% CI)	P-value
Fetal cardiac activities				
Absent	10.20 (1.93, 53.79)	0.006	-	-
Present	Ref			

At univariable analysis, fetal cardiac activities were found to be significantly associated with first-dose MTX treatment success. For variable selection, two additional variables were included (yolk sac and history of previous miscarriage). The final model of multivariable logistic regression analysis indicated that only cardiac activities were significantly associated with first-dose MTX treatment success. The absence of fetal cardiac activities was found to increase the likelihood of first-dose MTX treatment success (OR=10.20; 95% CI: 1.93, 53.79; P=0.006).

## Discussion

All previously published protocols for MTX treatment of ectopic pregnancies have restricted treatment to pre-treatment serum β-hCG levels - usually less than 5000 to 10,000 IU/L [19,20]. There is an inverse association between β-HCG levels and the successful medical management of an ectopic pregnancy. A systematic review by Menon et al. confirmed a substantial increase in the failure of medical management of ectopic pregnancy with single-dose MTX when the initial β-HCG level is above 5000 IU/L [19].

Serum β-hCG level showed a linear relationship with gestational sac size; thus, the level could be more than 5000 IU/L, and even more than 100,000 IU/L. In such cases, the patient was closely monitored as a tubal pregnancy case and recruited into the study after counseling, provided the patient was hemodynamic stable, agreed to MTX treatment, and was willing to attend follow-up appointments.

Seventy-three patients who received first-line MTX treatment for ectopic pregnancies were included in this study. Our overall success rate of 87.7% (76.7% response to the first dose and 11.0% response to the second dose) compares favorably with the results from other published studies, which range from approximately 65% to 95% (weighted mean 82%) [21-23]. A second dose of MTX was required in eight (11%) patients because of insufficiently declining serum β-hCG levels after a single dose of MTX. The single-dose protocol is the most commonly used because it is associated with fewer days of hospitalization and has fewer side effects. Most patients who failed MTX treatment underwent an operation because of their worsening clinical condition.

The presence of fetal cardiac activity or free fluid at the POD is often considered a relative contraindication to MTX treatment [9]. However, most of these restrictions are based on limited evidence. In this study, a higher pre-treatment serum β-hCG level is not the risk factor linked to the failure of treatment, but it is definitely associated with requiring more than one dose of MTX. Unlike previously published studies, we found that the smaller size of ectopic pregnancy and the absence of free fluid at POD increased the likelihood of treatment success [9,24]. The absence of fetal cardiac activities was the only independent predictor for second-dose MTX success.

All of the factors found to affect treatment success were related to the overall health of the ectopic conceptus. A high serum β-hCG level is associated with an ectopic conceptus still developing and growing. The presence of fetal cardiac activity indicates enough vascular support for pregnancy to continue into a more advanced stage of gestation. Thus, a lower serum β-hCG level or absence of fetal cardiac activity may indicate a very early or a failing ectopic pregnancy. MTX treatment is more effective with an early or failing ectopic pregnancy than with a more advanced ectopic pregnancy.

Ectopic pregnancies, especially in atypical sites, such as cesarean scar and cornual and cervical sites, are less well managed by systemic MTX and surgery. Thus, medical therapy with MTX injected directly into the ectopic gestational sac under ultrasound guidance has been advocated for these cases, and it has been proven to be a safe and effective alternative to surgical and systemic medical therapy [25]. In this study, we found that ultrasound-guided, directly injected MTX into the ectopic gestational sac successfully resolved the ectopic pregnancy in 15 out of 21 cases (71%). However, the success of the procedure varied according to the site of the ectopic pregnancy.

This study showed that the absence of fetal cardiac activity is the best prognostic indicator of treatment success in patients with ectopic pregnancies who are treated with a single-dose MTX protocol. We also speculated that the higher effectiveness of the local MTX treatment for atypical ectopic pregnancies might be due to the surrounding fibrous tissue and impaired vascularization, thus limiting the systemic diffusion of the drug to the pregnancy while favoring local administration. While the speculation is reasonable, a comparison of treatment methodology (direct or systemic) was not performed, limiting the integrity of the assumption.

The limitation of this study lies in the fact that a small number of cases was included, and this may not be generalized to the outcome of medical management of ectopic pregnancy. Furthermore, this retrospective review study may not represent the general population and is prone to selection bias. Further study is required, preferably prospective studies involving a large number of samples from multicenter hospitals in Malaysia.

## Conclusions

In conclusion, we reviewed our 10 years of experience treating ectopic pregnancies via medical therapy with MTX. While recognizing that our study was limited to 73 patients, the results of our study support the use of single-dose MTX as a first-line treatment for ectopic pregnancy in carefully selected patients. With further development of this study, we hope to create a local database on current medical therapy with MTX for treating ectopic pregnancy. Furthermore, the results of this study, which indicate the single-dose MTX protocol is successful, could be used to counsel and encourage eligible patients with ectopic pregnancies to undergo this treatment instead of surgery.
